# Distinctive accumulation of globotriaosylceramide and globotriaosylsphingosine in a mouse model of classic Fabry disease

**DOI:** 10.1016/j.ymgmr.2022.100952

**Published:** 2023-01-02

**Authors:** Atsumi Taguchi, Satoshi Ishii, Mariko Mikame, Hiroki Maruyama

**Affiliations:** a Department of Clinical Nephroscience, Niigata University Graduate School of Medical and Dental Sciences, Niigata, Japan; b Department of Matrix Medicine, Faculty of Medicine, Oita University, Oita, Japan; c Biochemical Laboratory, GlycoPharma Corporation, Oita, Japan

**Keywords:** Globotriaosylsphingosine, Globotriaosylceramide, Analog, Fabry disease, *Gla*^*tm*^*Tg(CAG-A4GALT)* Fabry mouse model, FD, Fabry disease, GLA, α-galactosidase A, Gb3, globotriaosylceramide, lyso-Gb3, globotriaosylsphingosine, ERT, Enzyme replacement therapy, PCT, pharmacological chaperone therapy, MeOH, methanol, ACN, acetonitrile, IPA, isopropyl alcohol, FA, formic acid, UPLC-MS/MS, ultra-performance liquid chromatography coupled to tandem MS.

## Abstract

Fabry disease (FD) is an inherited disease caused by deficient α-galactosidase A activity that is characterized by the accumulation of globotriaosylceramide (Gb3) and globotriaosylsphingosine (lyso-Gb3). Although plasma lyso-Gb3 is a sensitive biomarker of FD, the correlation between its concentration and clinical symptoms remains unclear. To clarify the influence of plasma Gb3 and lyso-Gb3 in a symptomatic *Gla*^*tm*^*Tg(CAG-A4GALT)* FD mouse model, the total contents of Gb3, lyso-Gb3 and their analogs in various organs and plasma were determined in mice with early- (5-week-old) and late-stage (20-week-old) renal dysfunction. A marked increase in total Gb3 content in the heart, kidneys, spleen, liver, small intestine, lungs, brain, and plasma was observed in the 20-week-old mice compared to that in 5-week-old mice. In contrast, the increase in lyso-Gb3 was relatively small, and the total content in the lungs and plasma was unchanged. Lyso-Gb3 analogs {lyso-Gb3(−2) and lyso-Gb3(+18)} and Gb3 analogs {Gb3(−2) and Gb3(+18)} were observed in all organs and plasma at both ages, and the percentages of the analogs were unique to specific organs. The pattern of 37 Gb3 analogs/isoforms of liver Gb3 corresponded well with that of plasma Gb3. Although the analog pattern of plasma lyso-Gb3 did not resemble that of any organ lyso-Gb3, the relative content {lyso-Gb3: lyso-Gb3(−2)} in the sum of all organs corresponded well to that of the plasma at both ages. These data indicate that liver Gb3 may contribute to the plasma Gb3 level, while plasma lyso-Gb3 may be released from all organs, and the capacity of the plasma lyso-Gb3 pool may reach a maximum at an early stage of renal dysfunction.

## 1 Introduction

Fabry disease (FD) is an X-linked inherited disorder caused by the deficient activity of a lysosomal hydrolase, α-galactosidase A (GLA, EC 3.2.1.22) [1]. FD can be classified as classic, which is distinguished by early onset classic manifestations of acroparesthesia, clustered angiokeratoma, cornea verticillata, and cerebrovascular and pulmonary involvements, or later-onset [2], which presents exclusively as cardiac, and renal impairments. Enzyme replacement therapy (ERT) [3,4] and pharmacological chaperone therapy (PCT) [5] are available for patients with FD. Although many positive results have been reported using both treatments, clinical outcomes should be determined individually, since anti-drug antibodies with neutralizing activities reduce the ERT effect [6], and the PCT effect is dependent on the mutation type [7,8]. To follow up on patients during treatment, a biomarker that predicts a clinical outcome is required.

Plasma globotriaosylceramide (Gb3) has been used as a biomarker to determine ERT efficacy [9] because it is the most abundant and common accumulation glycosphingolipid in FD. Plasma globotriaosylsphingosine (lyso-Gb3), which is a deacylated form of Gb3, is often used as a biomarker for the diagnosis and follow up of patients during treatment [10], even though plasma lyso-Gb3 levels are much lower than those of plasma Gb3. Plasma lyso-Gb3 is a more sensitive marker that has been used to diagnose later-onset and heterozygous female patients, which is not possible with plasma Gb3 [11]. Plasma lyso-Gb3 has high diagnostic sensitivity, and its level is correlated with phenotype [12]. However, there is no correlation between plasma lyso-Gb3 levels and clinical manifestations with PCT [13]. This discrepancy may be caused by a lack of knowledge regarding the origin of plasma lyso-Gb3.

Recently, many Gb3 isoforms (various fatty acids) and lyso-Gb3 analogs (various sphingolipid modifications) have been found in the plasma and urine of FD patients [14,15]. The presence of Gb3 isoforms and two lyso-Gb3 analogs {lyso-Gb3(−2) and lyso-Gb3(+18)} has been described in *GLA*-knockout mice [16]. We established a deacylation method for the total Gb3 concentration and the distribution of Gb3 analogs, and reported an organ-specific distribution of Gb3 isoforms and analogs/isoforms in our FD model *Gla*^*tm*^*Tg(CAG-A4GALT)* mice, whereby Gb3(+18) levels were high in the kidneys and liver, while Gb3(−2) levels were increased in the heart [17]. This technique is useful for the characterization of plasma Gb3 and lyso-Gb3.

To study the pathophysiology of FD, we previously generated a symptomatic *Gla*^*tm*^*Tg(CAG-A4GALT)* FD mouse model with renal impairment and demonstrated that increased Gb3 accumulation caused this condition. *Gla*^*tm*^*Tg(CAG-A4GALT)* mice showed progressive renal impairment, and premature death occurred after 35 weeks of age [18]. This mouse line is a classic FD model due to the complete loss of GLA activity. We have previously demonstrated that dysfunction of the medullary thick ascending limbs leads to polyuria [19], and excess urinary excretion of divalent cations by 20 weeks of age causes accelerated bone resorption and osteomalacia [20].

The purpose of the present study was to clarify changes in the accumulation of Gb3 and lyso-Gb3 and their analogs by comparing the levels before (5-week-old) and after (20-week-old) the decline in renal function, so that we might understand the pathogenic role of organ and plasma Gb3 and lyso-Gb3 and their analogs. We measured Gb3, lyso-Gb3 and their analogs in the major organs (heart, kidneys, spleen, liver, small intestine, lungs, and brain) and plasma. The heart, kidneys, small intestine, and brain are susceptible to FD [21], and the heart, kidneys, and brain are also associated with a poorer prognosis and resistance to ERT. In contrast, the spleen, liver, and lungs are not susceptible to FD, but the treated enzymes are markedly transferred to the liver [22]. By comparing organs and plasma, we discuss what we can learn from the plasma Gb3 and lyso-Gb3 levels.

## 2 Materials and methods

### 2.1 Chemicals and reagents

HPLC-grade methanol (MeOH), distilled water (H_2_O), acetonitrile (ACN), isopropyl alcohol (IPA), formic acid (FA), analytical-grade chloroform (CHCl_3_), hexane, and other reagents were obtained from FUJIFILM Wako Pure Chemical Corporation (Osaka, Japan). Iatrobeads were obtained from Iatron Laboratories (Tokyo, Japan). Standard Gb3 (porcine RBC) was purchased from Matreya LLC (State College, PA, USA). Standard lyso-Gb3 and a glycine derivative of lyso-Gb3 (Gly-lyso-Gb3) were prepared as previously described [23].

### 2.2 Animals

The male *Gla*^*tm*^*Tg(A4GALT)* mice used in the present study were housed under standard laboratory conditions at the animal facility of Niigata University. The experiments were conducted according to the principles and procedures outlined in the Science Council of Japan's Guidelines for Proper Conduct of Animal Experiments and were approved by the presidents of Niigata University (SA00386, SA00877, SD01036, and SD01487). Approximately 0.1 g of each organ from 5-week-old and 20-week-old *Gla*^*tm*^*Tg(CAG-A4GALT)* mice was collected and transferred to Oita University for the determination of Gb3 and lyso-Gb3 content. Heparinized plasma samples were separated by centrifugation at 3000 ×*g* for 10 min, and all samples were stored at −20 °C. The organs were homogenized in a 10-fold volume of H_2_O, and the protein concentration of the homogenates was assayed using the protein assay rapid kit *Wako* II (FUJIFILM Wako Pure Chemical Corp.).

### 2.3 Total lyso-Gb3 and Gb3 assays

The total contents of lyso-Gb3 (lyso-Gb3 and its analogs) and Gb3 (Gb3 and its analogs/isoforms) in mouse organs and plasma were determined as described previously [17] with the following modifications. Aliquots (40 μl) of organ homogenates (protein concentration: 1 mg/ml) and plasma samples were mixed with 0.8 ml of CHCl_3_/MeOH (2:1 [*v*/v]) and 20 μl of 40 ng/ml Gly-lyso-Gb3 (as an internal standard); aqueous lipids were extracted by the addition of 0.2 ml of 1.5% FA in H_2_O; and centrifugation was performed at 6500 ×*g* using a personal centrifuge (Gyrogen, Seoul, Korea) for 5 min. The upper layer lyso-Gb3 samples (200 μl) were dried using a centrifugal evaporator CVE-2000 (EYELA, Tokyo, Japan), re-dissolved in 50 μl H_2_O, and extracted with the same volume of H_2_O-saturated 1-butanol. The extracts were diluted with MeOH and analyzed using ultra-performance liquid chromatography coupled to tandem MS (UPLC-MS/MS). The peak areas corresponding to lyso-Gb3 and its analogs in the multiple reaction monitoring chromatogram were calculated using MassLynx software (Waters).

The lower layer Gb3 samples (40 μl for 5-week mouse organs or 4 μl for 20-week mouse organs) were dried, suspended in 40 μl of enzyme mixture {5 μg/ml of sphingolipid ceramide N-deacylase (kindly supplied by Dr. Okino, Kyusyu University) in 25 mM sodium acetate buffer (pH 5.5) containing 0.5% Triton X-100, 5 mM CaCl_2_, and 5% dimethyl sulfoxide}, and incubated at 37 °C for 1 h. The reaction was stopped by the addition of 0.8 ml CHCl_3_/MeOH (2:1 [*v*/v]). The reaction products were mixed with 20 μl of 100 ng/ml Gly-lyso-Gb3 and 0.2 ml of 1.5% FA in H_2_O and extracted by centrifugation at 6500 ×*g* for 5 min. Subsequently, 125 μl of the clear upper layer was transferred to another tube and dried in a centrifugal evaporator. The samples were redissolved in 50 μl H_2_O and extracted with the same volume of H_2_O-saturated 1-butanol. The extracts were diluted with MeOH and analyzed using UPLC-MS/MS.

### 2.4 Direct assay of Gb3 analogs/isoforms

The patterns of 37 Gb3 analogs/isoforms were determined using a direct assay, as described previously [17]. Aliquots (20 μl) of tissue homogenates (1 mg/ml or 0.1 mg/ml for 5-week or 20-week-old mice, respectively) and plasma were mixed with 0.4 ml of CHCl_3_/MeOH (2:1 [*v*/v]) for lipid extraction. After addition of 20 μl of internal standard Gb3(d18:2)(C17:0) (40 ng/ml), aqueous lipids were separated by the addition of 0.1 ml of 1.5% FA in H_2_O and centrifuged at 6500 ×*g* for 5 min. After removing the upper layer, 40 μl of the lower layer was transferred to another tube and dried under a stream of nitrogen. Dried samples were subjected to mild alkaline treatment with 200 μl of 0.2 N NaOH in MeOH at 40 °C for 1 h. After neutralizing the solution with glacial acetic acid, 400 μl of CHCl_3_ and 150 μl of H_2_O were added to the sample solution, and glycolipids were extracted by centrifugation at 6500 ×*g* for 5 min. After the removal of the upper layer, 160 μl of MeOH and 300 μl of hexane were added to the lower layer. Samples were placed in an Iatrobeads column (0.5 × 1 cm) equilibrated with hexane. After washing the column with 0.6 ml of IPA-hexane (50:50 [*v*/v]), the bound glycolipids were eluted with 0.6 ml of IPA-hexane-H_2_O (50:35:15 [v/v/v]). Eluates were pooled, dried, dissolved in 0.1 ml of MeOH, and analyzed by UPLC-MS/MS, as described previously [17].

### 2.5 Statistical analysis and graph preparation

At least six mice/group were used in all studies. Two-tailed significance values are reported. We used the Shapiro-Wilk test to test for a normal distribution. Normally distributed data were evaluated for variance using the *F*-test. Statistical analyses were performed using the student's *t*-test, Welch's *t*-test, and Wilcoxon rank-sum test with JMP®12 software (SAS Institute, Cary, NC, USA). Statistical significance was set at *p*-value <0.05.

## 3 Results

### 3.1 Total Gb3 and lyso-Gb3 in major organs of Gla^tm^Tg(CAG-A4GALT) mice

**Fig. 1:**
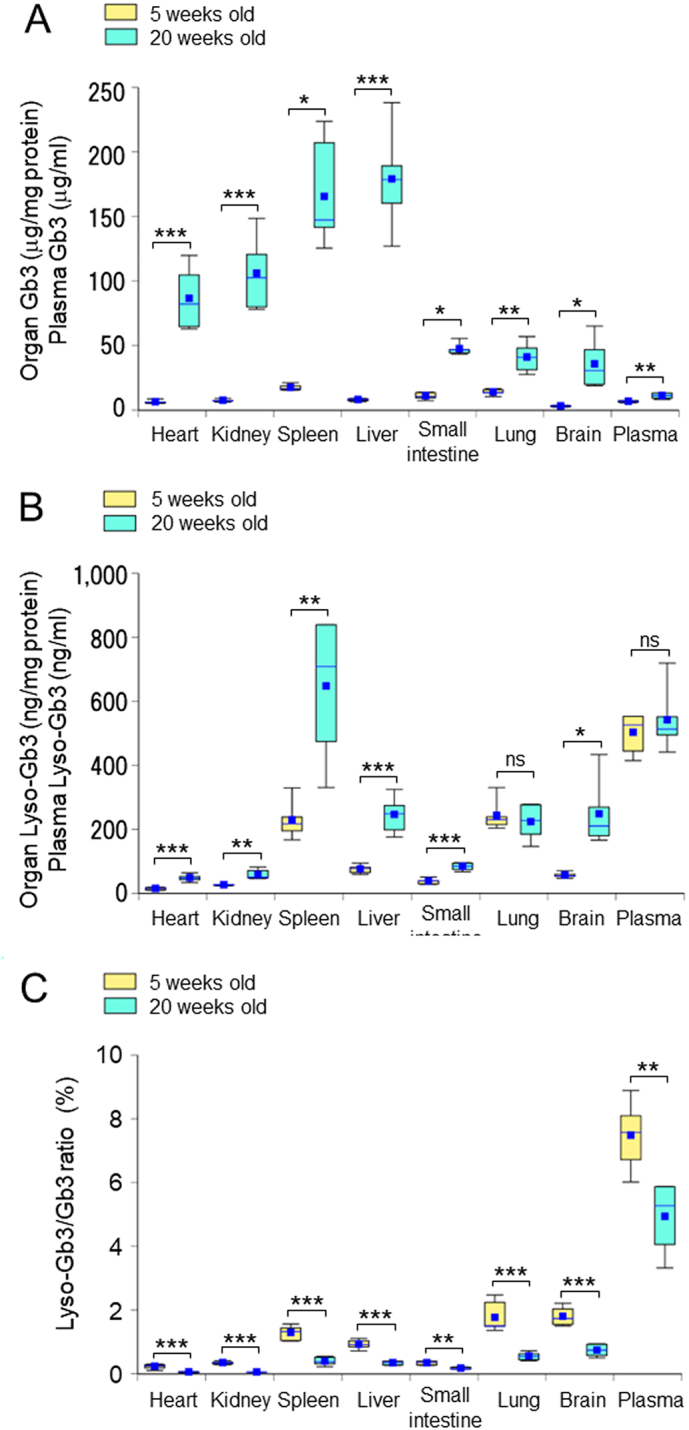
Total content of Gb3 and lyso-Gb3 in major organs and plasma. (**A**) Total Gb3 (Gb3 isoforms and Gb3 analogs/isoforms) in seven organs and plasma from 5- and 20-week-old *Gla*^*tm*^*Tg(CAG-A4GALT)* mice (*n* = 6 / group) was determined by the deacylation method. Organ Gb3 and plasma Gb3 is expressed as μg/mg protein and μg/ml, respectively. (**B**) Total lyso-Gb3 (lyso-Gb3 and its analogs) in seven organs and plasma from 5- and 20-week-old *Gla*^*tm*^*Tg(CAG-A4GALT)* mice (*n* = 6 / group) was assayed as described in the Materials and methods section. Organ and plasma lyso-Gb3 is expressed as ng/mg protein and ng/ml, respectively. (**C**) The percentage of lyso-Gb3/Gb3 was calculated from the data of **A** and **B**. In box-and-whisker plots, center lines represent the median, box limits represent quartiles, whiskers represent the 10th and 90th percentiles, and blue dots represent the means. Differences between 5- and 20-week-old mice were evaluated as described in the Materials and methods section. * *p*-value <0.05; ** *p*-value <0.01; *** *p*-value <0.001; ns, not significant.

The total content (including each analog and isoform) of Gb3 and lyso-Gb3 in major organs and plasma from 5-week and 20-week-old *Gla*^*tm*^*Tg(CAG-A4GALT)* mice is summarized in Fig. 1. Substantial increases were observed with age in organ Gb3 levels, including 13.5-, 14.1-, 9.5-, 21.8-, 4.3-, 2.9-, and 11.2-fold increases in the heart, kidneys, spleen, liver, small intestine, lungs, and brain, respectively. In contrast, the increase in organ lyso-Gb3 content was relatively small, with 3.3-, 2.2-, 2.8-, 3.2-, 2.2-, and 4.4-fold increases in the heart, kidneys, spleen, liver, small intestine, and brain, respectively, and no increase was observed in the lungs. In the plasma, Gb3 was slightly greater (1.6-fold), but no change was observed in lyso-Gb3 content.

A relatively low percentage of lyso-Gb3/Gb3 (approximately 0.3%) was observed in the heart, kidneys, and small intestine of 5-week-old mice, and these percentages were further decreased at 20 weeks of age. The lowest percentage of lyso-Gb3/Gb3 (0.06%) was noted in the heart and kidneys of the 20-week-old mice. The percentage of lyso-Gb3/Gb3 was high in the plasma at both ages (7.5% and 3.4% at 5 and 20 weeks of age, respectively).

### 3.2 Distribution of lyso-Gb3 and its analogs in major organs and plasma

**Fig. 2:**
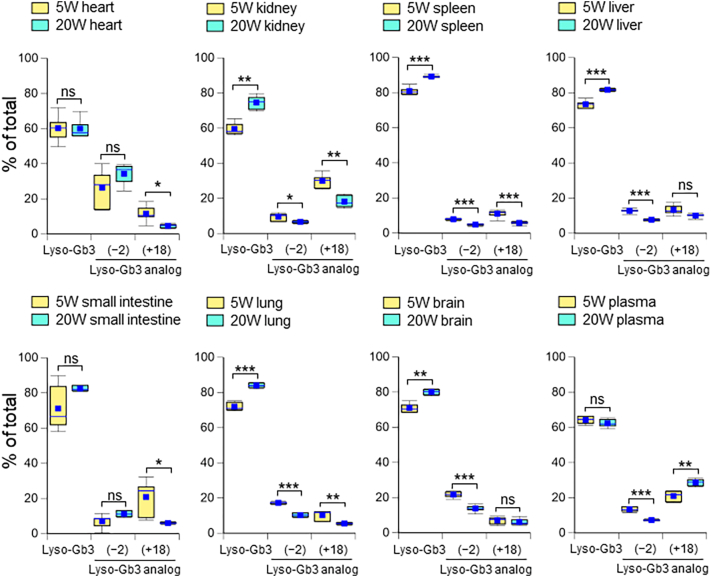
Distribution of lyso-Gb3 and its analogs in major organs and plasma. Distribution of lyso-Gb3 and its analogs {lyso-Gb3(−2) and lyso-Gb3(+18)} were determined as the percentage of total lyso-Gb3 content in organs and plasma from 5- and 20-week-old *Gla*^*tm*^*Tg(CAG-A4GALT)* mice (*n* = 6 / group). In box-and-whisker plots, center lines represent the median, box limits represent quartiles, whiskers represent the 10th and 90th percentiles, and blue dots represent the means. Differences between 5- and 20-week-old mice were evaluated as described in the Materials and methods section. * *p*-value <0.05; ** *p*-value <0.01; *** *p*-value <0.001; ns, not significant.

Next, we investigated whether the distribution of lyso-Gb3 and its analogs in *Gla*^*tm*^*Tg(CAG-A4GALT)* mouse organs changed with age. Although we discerned lyso-Gb3 and its eight analogs, we have only described the distribution of lyso-Gb3 and two major lyso-Gb3 analogs {lyso-Gb3(−2) and lyso-Gb3(+18)} because >98% of the total concentration in *Gla*^*tm*^*Tg(CAG-A4GALT)* mouse organs and plasma was occupied by these compounds (Fig. 2). The percentage of lyso-Gb3 increased in six organs with age, but those in the heart and plasma were unchanged. The distribution of lyso-Gb3(+18) in all seven organs decreased and increased in the plasma with age. No pattern of plasma lyso-Gb3 and its analogs was observed in the organ lyso-Gb3 and its analogs.

### 3.3 Distribution of Gb3 and its analogs in major organs and plasma

**Fig. 3:**
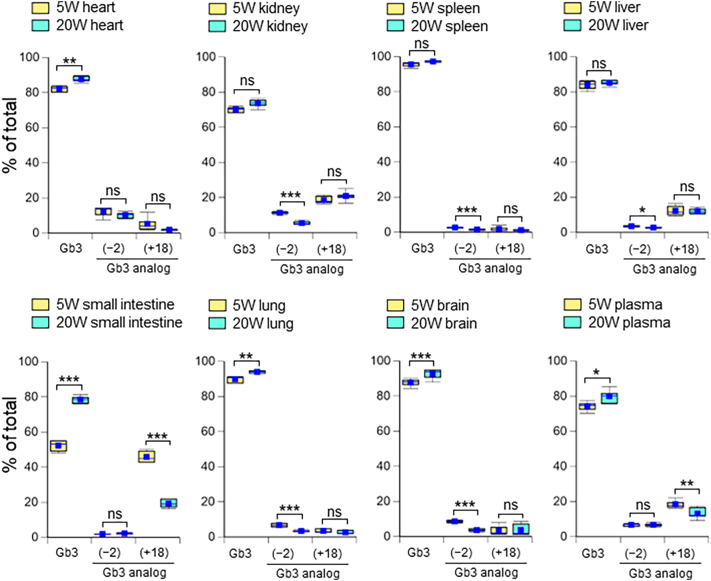
Distribution of Gb3 isoforms and Gb3 analogs/isoforms in major organs and plasma. Distribution of Gb3 isoforms and Gb3 analogs/isoforms {Gb3(−2) and Gb3(+18)} were determined as the percentage of total Gb3 content in organs and plasma from 5- and 20-week-old *Gla*^*tm*^*Tg(CAG-A4GALT)* mice (*n* = 6 / group). In box-and-whisker plots, center lines represent the median, box limits represent quartiles, whiskers represent the 10th and 90th percentiles, and blue dots represent the means. Differences between 5- and 20-week-old mice were evaluated as described in the Materials and methods section. * *p*-value <0.05; ** *p*-value <0.01; *** *p*-value <0.001; ns, not significant.

The changes in the Gb3 analog distributions with age were relatively small but similar to those of the lyso-Gb3 analog distributions (Fig. 3). The presence of Gb3 analogs in the heart, kidneys, and liver was detected as described in our previous paper [17]. We observed a high distribution of Gb3(+18) (43.8% of total Gb3) in the small intestine of 5-week-old *Gla*^*tm*^*Tg(CAG-A4GALT)* mice. The pattern of Gb3 and its analogs in plasma Gb3 was similar to that of liver Gb3 at both ages.

### 3.4 Profile of Gb3 isoforms and Gb3 analogs/isoforms in major organs and plasma

**Fig. 4:**
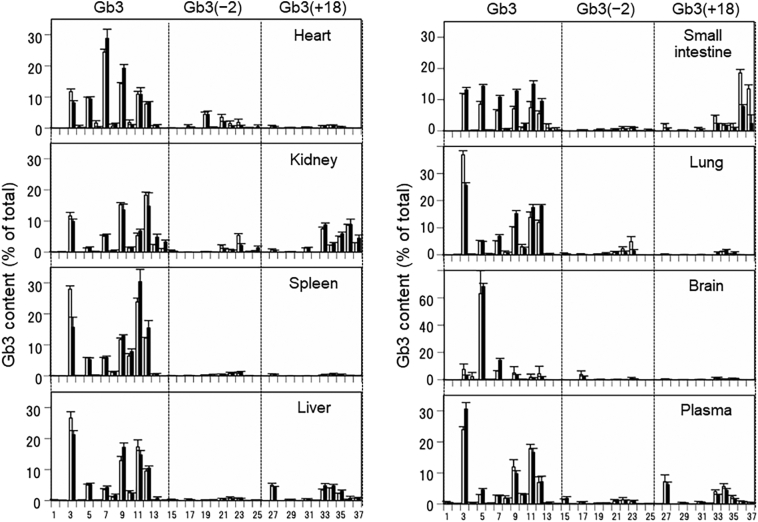
Gb3 profiles in major organs and plasma. Gb3 extracted from organs and plasma were applied to UPLC-MS/MS and determined by the direct assay as described in the Materials and methods section. Results are means ± S.D. (error bars) of organ homogenates from 5-week-old (white bar) and 20-week-old (black bar) *Gla*^*tm*^*Tg(CAG-A4GALT)* mice (*n* = 4 / group). 1, Gb3(d16:1)(C16:0); 2, Gb3(d18:1)(C16:1); 3, Gb3(d18:1)(C16:0); 4, Gb3(d18:1)(C18:1); 5, Gb3(d18:1)(C18:0); 6, Gb3(d18:1)(C20:1); 7, Gb3(d18:1)(C20:0); 8, Gb3(d18:1)(C22:1); 9, Gb3(d18:1)(C22:0); 10, Gb3(d18:1)(C24:2); 11, Gb3(d18:1)(C24:1); 12, Gb3(d18:1)(C24:0); 13, Gb3(d18:1)(C22:0-OH); 14, Gb3(d18:1)(C24:0-OH); 15, Gb3(d18:2)(C16:0); 16, Gb3(d18:2)(C18:1); 17, Gb3(d18:2)(C18:0); 18, Gb3(d18:2)(C20:1); 19, Gb3(d18:2)(C20:0); 20, Gb3(d18:2)(C22:1); 21, Gb3(d18:2)(C22:0); 22, Gb3(d18:2)(C24:1); 23, Gb3(d18:2)(C24:0); 24, Gb3(d18:2)(C22:0-OH); 25, Gb3(d18:2)(C24:0-OH); 26, Gb3(d18:0-OH)(C16:1); 27, Gb3(d18:0-OH)(C16:0); 28, Gb3(d18:0-OH)(C18:1); 29, Gb3(d18:0-OH)(C18:0); 30, Gb3(d18:0-OH)(C20:1); 31, Gb3(d18:0-OH)(C20:0); 32, Gb3(d18:0-OH)(C22:1); 33, Gb3(d18:0-OH)(C22:0); 34, Gb3(d18:0-OH)(C24:1); 35, Gb3(d18:0-OH)(C24:0); 36, Gb3(d18:0-OH)(C22:0-OH); 37, Gb3(d18:0-OH)(C24:0-OH).

To determine the aging effect of the heterogeneity of Gb3 isoforms and Gb3 analogs/isoforms, we compared the profiles of the 37 Gb3 isoforms and Gb3 analogs/isoforms from organs of 5- and 20-week-old mice (Fig. 4). Organ-specific Gb3 profiles were observed, and no identical Gb3 profiles were detected in the seven organs. The plasma Gb3 profile corresponded well with that of the liver. Although a considerable increase in Gb3 content occurred during aging, the basic profiles in all seven organs and the plasma did not change. Smaller changes were observed; for example, the Gb3 isoform with shorter chain fatty acid Gb3(d18:1)(C16:0) decreased with age in six organs and increased in the plasma. The aging effect was most obvious in the small intestine, where the Gb3 isoforms with saturated fatty acids were increased and the Gb3(+18) analog/isoforms with hydroxy fatty acids were decreased.

## 4 Discussion

### 4.1 Changes in total GSL content in between 5- and 20-week-old Gla^tm^Tg(CAG-A4GALT) mice

*Gla*^*tm*^*Tg(CAG-A4GALT)* mice show progressive renal impairment [19]. Although 5-week-old *Gla*^*tm*^*Tg(CAG-A4GALT)* mice did not present renal dysfunction, significant accumulation of Gb3 in the kidneys was detected in comparison with age-matched wild-type mice. The Gb3 level in organs of 5-week-old *Gla*^*tm*^*Tg(CAG-A4GALT)* mice was similar to that in *GLA*-knockout mice, but a difference was noted after 10 weeks of age [18]. Therefore, Gb3 accumulation during this period may indicate the crucial point for pathogenic onset. We chose 5- and 20-week-old FD mice with early- and late-stage renal dysfunction, and determined the contents of organ Gb3 and lyso-Gb3, and their plasma concentrations. The total Gb3 content increased markedly with age, as previously described [18]. In contrast, the fold-increase in the total lyso-Gb3 content was relatively small. Gb3 easily accumulates in organs because the chemical property of Gb3 is more hydrophobic than lyso-Gb3. It is possible that the production of lyso-Gb3 is a method to reduce the accumulation of Gb3 in a GLA-deficient body [24]. The percentage of lyso-Gb3/Gb3 in plasma observed in the present study was much higher than that in organs, which may indicate the secretion pathway of accumulated Gb3. An *in vitro* study suggested that lyso-Gb3 is produced from Gb3 by the enzymatic deacylation of acid ceramidase [24]. Although further study is required on the mechanism of lyso-Gb3 production, it is interesting that a low level of transition from Gb3 to lyso-Gb3 was observed by the low percentage of lyso-Gb3/Gb3 in FD-related organs such as the heart, kidneys, and small intestine. Renal impairment in *Gla*^*tm*^*Tg(CAG-A4GALT)* mice may have been caused by the substantial accumulation of Gb3 (14.1-fold increase during this period) in the kidneys; however, the effect of lyso-Gb3 may be limited due to the low level of increase (2.2-fold).

### 4.2 Comparison of GSL profiles between organs and plasma

Recently, plasma lyso-Gb3 has been used as a biomarker for FD diagnosis [25,26] and follow-up of patients during treatment [11,27,28]. However, the concentration of plasma lyso-Gb3 did not correlate with the accumulation of Gb3 in organs from classic FD model mice (Fig. 1). It may not be possible to ascertain organ Gb3 accumulation in the plasma samples of classic FD patients. Although plasma lyso-Gb3 is not correlated with organ lyso-Gb3, it is useful for the diagnosis of patients prior to renal dysfunction because plasma lyso-Gb3 levels are high despite low Gb3 accumulation in the organ. Therefore, this may lead to early diagnosis and treatment, which is important for effective clinical outcomes [29]. We reported the organ-specific Gb3 profile in the heart, kidneys, spleen, and liver in a previous study [17], and we have further observed unique Gb3 profiles in the small intestine, lungs, and brain. None of these were identical, but the plasma Gb3 profile was similar to that of the liver (Fig. 4).

### 4.3 Absolute organ Gb3 content

**Fig. 5:**
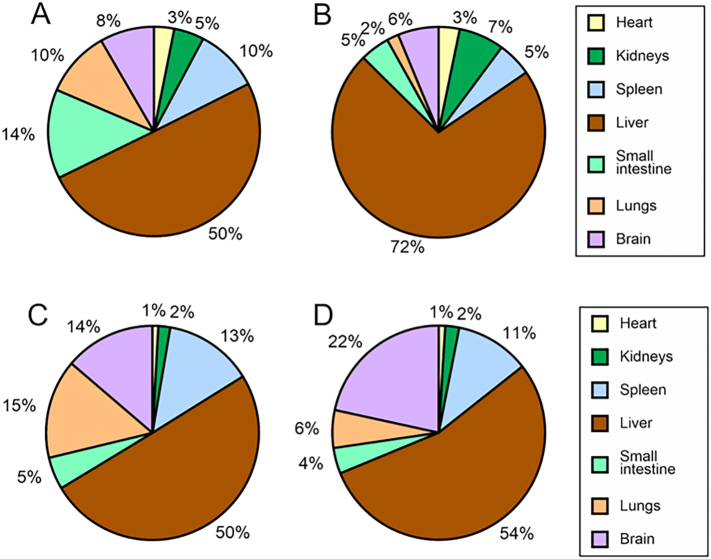
Percentage of absolute organ content of total Gb3 and lyso-Gb3 in 5-week-old and 20-week-old *Gla*^*tm*^*Tg(CAG-A4GALT)* mice. Absolute Gb3 and lyso-Gb3 accumulated in each organ (GSL/organ) were calculated from the concentration (GSL/mg protein) with organ protein content (mg protein/organ). The mean values of their concentrations described in Fig. 1 was used. The mean value of protein contents of the heart, kidneys, spleen, liver, small intestine, lungs and brain from 5-week-old mice were 17.0, 21.8, 18.6, 204.3, 46.3, 21.9 and 83.5 mg/organ, respectively, and those from 20-week-old mice were 22.5, 39.3, 19.1, 241.7, 54.8, 28.4 and 84.2 mg/organ, respectively. Total Gb3 in organs from 5-week-old mice is described in A, and that from 20-week-old mice in B. C, total organ lyso-Gb3 in 5-week-old mice; and D, total organ lyso-Gb3 in 20-week-old mice.

To discuss the influence of plasma Gb3 and lyso-Gb3, the total content in each organ (Gb3/organ) was calculated from the concentration (Gb3/mg protein) with organ protein content (mg protein/organ), and the relative contents of total Gb3 and lyso-Gb3 in seven organs are shown in Fig. 5. The liver contained the highest levels of both Gb3 and lyso-Gb3 at both ages. Although the accumulation profile of lyso-Gb3 did not change with age, liver Gb3 levels markedly increased with age, from 50% at 5 weeks to 72% at 20 weeks of age. In the present study, we found that the Gb3 profile in the plasma was in good agreement with that in the liver. Plasma Gb3 has been associated with lipoproteins [10], which indicates that it originated mainly in the liver. The selective accumulation of Gb3 in the liver may be caused by a decrease in its secretion from this organ with age, though we have no experimental data on the Gb3 secretion from organs.

### 4.4 Comparison between organ lyso-Gb3 and plasma lyso-Gb3

**Fig. 6:**
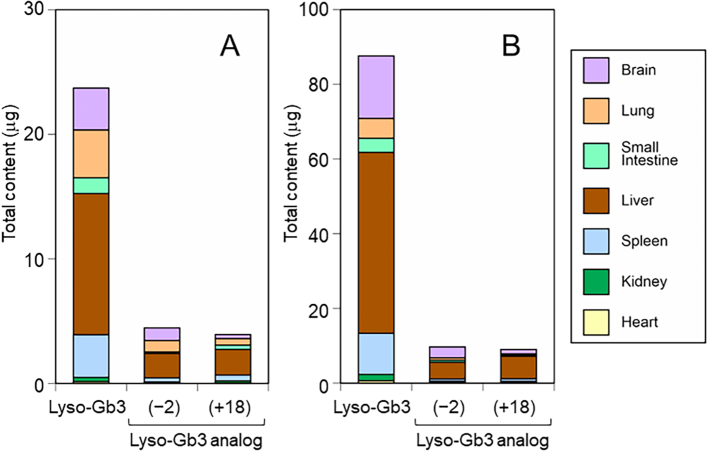
Sum of individual content of organ lyso-Gb3 and its analogs. The absolute individual lyso-Gb3 and its analogs were calculated as described in the legend of Fig. 5. The sum of individual lyso-Gb3 and its analogs in seven organs from 5-week-old mice is presented in A and those from 20-week-old mice in B.

In contrast to Gb3, all organ lyso-Gb3 may equally contribute to the level of plasma lyso-Gb3 because of its greater hydrophilic properties compared to Gb3. To test this hypothesis, we studied the correlation between the lyso-Gb3 profile between the total organs (Fig. 6) and plasma (Fig. 2). Relative concentrations (lyso-Gb3 as 100%) of organ lyso-Gb3(−2) at 5 and 20 weeks of age were 18.8% and 11.1%, respectively, which corresponded well to those of plasma (20.8% and 11.5%, respectively). The relative concentrations of organ lyso-Gb3(+18) at 5 and 20 weeks of age (16.5% and 10.3%, respectively) were much lower than those of plasma (32.7% and 45.0%, respectively). Although the chemical structure of lyso-Gb3(+18) is still unknown, it is more hydrophilic than lyso-Gb3 and lyso-Gb3(−2), and this may cause its higher flow rate from organs to plasma. Assuming that plasma lyso-Gb3 was released from organs equally, we can calculate the relative flow of lyso-Gb3 and its analogs from organs to plasma (Table 1). In comparison with 5-week-old mice, the accumulation of lyso-Gb3 and its analogs was markedly increased in the organs of 20-week-old mice, and the changes in the plasma levels in this period were smaller. Therefore, the relative flow from organs to plasma was decreased in 20-week-old mice, with identical reduction levels between lyso-Gb3 and lyso-Gb3(−2). From these data, we suggest that plasma lyso-Gb3 and its analogs may be secreted from all organs, while there may be a limit in the plasma lyso-Gb3 pool. The flow rate of lyso-Gb3 reached a maximum level at 5 weeks of age in our classic FD model mice because the total lyso-Gb3 concentration in plasma did not change after this point. Classic FD patients show high plasma lyso-Gb3 levels from an infant age; however, elevated plasma lyso-Gb3 levels could be leaked from the spleen and liver, which may not reflect the accumulation in clinically relevant organs such as the heart, kidneys, or peripheral nerves. Therefore, it may be difficult to follow up with plasma lyso-Gb3 levels in patients with classic FD.

**Table 1:** Relative flow of lyso-Gb3 and its analogs from organs to plasma in 20-week-old mice as compared to 5-week-old mice.

	Relative content (%) (20-week-old/5-week-old)		Relative flow
	Organ	Plasma	Plasma/Organ (%)
Lyso-Gb3	373	106	28
Lyso-Gb3(−2)	211	58	28
Lyso-Gb3(+18)	244	145	60

The relative content of lyso-Gb3 and its analogs in organs was calculated from the mean value of the total organ content from 20-week-old mice (Fig. 6B) compared to that of 5-week-old mice (Fig. 6A).

### 4.5 Limitations

This study had several limitations. The modification of lyso-Gb3 to lyso-Gb3(+18) may reduce the hydrophobicity of lyso-Gb3 and accelerate the excretion of these compounds, but it is unclear whether any regulatory system is present. In addition, the distribution of lyso-Gb3 analogs was often higher than that of Gb3 analogs in organs; however, the distribution of Gb3 analogs in the small intestine was higher than that of lyso-Gb3 analogs in 5-week-old *Gla*^*tm*^*Tg(CAG-A4GALT)* mice. Although we cannot yet explain this phenomenon, a unique synthesis of Gb3 analogs may be present in the small intestine. The correlation between Gb3 accumulation and pathogenesis in organs other than the kidneys cannot currently be discussed because the organ condition has not been fully observed, especially in relation to the heart and small intestine.

## 5 Conclusions

In the present study, we described the age-related and organ-specific accumulation of Gb3 and lyso-Gb3 in *Gla*^*tm*^*Tg(CAG-A4GALT)* mice. Drastic increase in the organ Gb3 content must be important on the pathogenesis of FD. We suggested that plasma Gb3 is correlated with liver Gb3, but plasma lyso-Gb3 may be secreted from all organs. Therefore, it may not be possible to determine Gb3 accumulation in FD-relevant organs from plasma Gb3 or lyso-Gb3 concentrations in patients with classic FD. Our symptomatic mouse model will be useful for preclinical studies on FD treatment, and our present data can help to understand the natural course of Gb3, lyso-Gb3 and their analogs accumulation for determining treatment efficacy. The presence of lyso-Gb3 analogs and Gb3 analogs should not be ignored, and the effect of the treatment on the total content of Gb3, lyso-Gb3 and their analogs must be determined.

## Authors' contributions

AT, SI, and HM conceived of and designed the study. AT, MM, and HM treated mice and collected the samples. SI determined the GSL concentrations. AT and SI wrote the manuscript. HM supervised the study. All authors have critically reviewed and revised the manuscript and accepted the final version.

## Funding

This work was funded by Sanofi K.K., Amicus Therapeutics K.K., JMS Co. Ltd., Terumo Corp., Torii Pharmaceutical Co. Ltd., and GlycoPharma Corp.

## Declaration of Competing Interest

H.M. received research support and speaker fees from Amicus Therapeutics K.K., Sanofi K.K., and Terumo Corp. and research support from Aoikai Medical Co., JCR Pharmaceuticals Co., Ltd., JMS Co., Ltd., and Torii Pharmaceutical Co., Ltd. S.I. is an employee and shareholder of GlycoPharma Corp. and received speaker fees from Sanofi K.K., Amicus Therapeutics K.K., and Sumitomo Dainippon Pharma Co., Ltd. The remaining authors declare no conflicts of interest.

## Data Availability

Data will be made available on request.
